# The impact of surgical intervention on renal function in cystinuria

**DOI:** 10.1590/2175-8239-JBN-2018-0034

**Published:** 2018-06-21

**Authors:** Serra Sürmeli Döven, Ali Delibaş, Hakan Taşkınlar, Ali Naycı

**Affiliations:** 1 Mersin University Faculty of Medicine, Department of Pediatric Nephrology, Mersin, Turkey.; 2 Mersin University Faculty of Medicine, Department of Pediatric Urology, Mersin, Turkey.

**Keywords:** Cystinuria, Urolithiasis, Surgery, Childhood, Renal Scarring, Cistinúria, Urolitíase, Cirurgia, Infância, Cicatrizes Renais

## Abstract

**Introduction:**

Cystinuria is an autosomal recessive disorder due to intestinal and renal transport defects in cystine and dibasic amino acids, which result in recurrent urolithiasis and surgical interventions. This study aimed to assess the impact of surgical interventions on renal function by analyzing estimated glomerular filtration rates.

**Methods:**

Thirteen pediatric patients with cystinuria, who were followed-up in a single tertiary institution between 2004 and 2016, were included in the study. Medical records were reviewed to collect data on clinical presentation of patients, urine parameters, stone formation, medical treatment, surgical intervention, stone recurrence after surgical procedure, stone analysis, ultrasonography, 99m-technetium dimercaptosuccinic acid (99mTc-DMSA) radionuclide imaging results, and follow-up time. Creatinine clearances estimated by modified Schwartz (eGFR) formula before and after surgery were used to assess renal function and compared statistically.

**Results:**

Nine patients (69.2%) had renal scarring which were detected with 99mTc-DMSA radionuclide imaging. In ten patients (76.9%), open surgical intervention for stones were needed during follow-up. Significant difference was not detected between eGFR before and after surgical intervention (mean 92 *versus* 106, *p* = 0.36). Nine of the patients (69.2%) were stone free in the last ultrasonographic examination. Relapses of stone after surgery were seen in 66.6% of patients who underwent surgical intervention.

**Conclusions:**

Surgical interventions for urinary stones are commonly required in patients with cystinuria. Renal scarring is a prevalent finding in cystinuric patients. Surgical interventions have no negative impact on eGFR in patients with cystinuria according to the present study.

## RESUMO

**Introdução:**

A cistinúria é um distúrbio autossômico recessivo causado por defeitos de transporte intestinal e renal da cistina e aminoácidos dibásicos que resultam em urolitíase recorrente e necessidade de intervenção cirúrgica. O presente estudo teve por objetivo avaliar o impacto das intervenções cirúrgicas sobre a função renal por meio da análise da taxa de filtração glomerular estimada.

**Métodos:**

Treze pacientes pediátricos com cistinúria acompanhados em uma instituição terciária entre 2004 e 2016 foram incluídos no estudo. Os prontuários médicos foram analisados e utilizados como fonte de dados sobre a apresentação clínica dos pacientes, parâmetros urinários, formação de cálculos, tratamento clínico, intervenção cirúrgica, recidiva de cálculos após procedimento cirúrgico, análise de cálculos, ultrassonografia, resultados de imagens com ácido dimercaptossuccínico marcado com tecnécio metaestável (99mTc-DMSA) e tempo de seguimento. A depuração de creatinina estimada pela fórmula modificada de Schwartz (TFGe) antes e após a cirurgia foi utilizada para avaliar e comparar estatisticamente os níveis de função renal.

**Resultados:**

Nove pacientes (69,2%) apresentaram cicatrizes renais detectadas por exame de imagem com 99mTc-DMSA. Dez pacientes (76,9%) necessitaram intervenção cirúrgica aberta por cálculo renal durante o seguimento. Não foram detectadas diferenças significativas entre os valores de TFGe anteriores e posteriores à intervenção cirúrgica (média de 92 *vs*. 106, *p* = 0,36). Nove pacientes (69,2%) não apresentaram cálculos no último exame ultrassonográfico. Recidivas de cálculos renais após cirurgia foram observadas em 66,6% dos pacientes submetidos a cirurgia.

**Conclusões:**

Intervenções cirúrgicas relativas a cálculos renais são frequentemente necessárias em pacientes com cistinúria. Cicatrizes renais são um achado prevalente em pacientes com cistinúria. De acordo com o presente estudo, cirurgia não afeta negativamente a TFGe de pacientes com cistinúria.

## INTRODUCTION

Cystinuria is an autosomal recessive disorder due to intestinal and renal transport defects in cystine and dibasic amino acids: arginine, lysine, and ornithine. Because of the poor solubility of cystine at the physiological pH of urine, patients have a lifelong risk of urinary stone formation^1^. If not diagnosed and treated properly, cystinuria results in recurrent urolithiasis with significant morbidity due to obstruction, infection, and repeated surgical intervention^2^. Renal impairment in patients with stone-forming cystinuria is a common finding^3^. However, there are few studies about the impact of surgical interventions on renal function in patients with cystinuria^4^.

In this study, we aimed to assess the renal outcomes of cystinuria and the impact of surgical interventions on renal function by analyzing the estimated glomerular filtration rates in patients with cystinuria followed in our institution.

## METHODS

A retrospective study was conducted in patients with cystinuria diagnosed, treated, and followed in our institution from January 2004 to August 2016. The Ethical Committee of the Mersin University Medical Faculty approved the study on 20/10/2016. Cystinuria diagnosis was based on quantitative urinary cystine excretion by amino acid chromatography in patients with nephrolithiasis. Medical records were reviewed to collect data on age of diagnosis, disease presentation, parental consanguinity, family history for stone disease, metabolic investigation in urine samples, stone formation, medical treatment, surgical intervention, stone recurrence after surgical procedure, stone analysis, ultrasonographic and scintigraphic investigations with 99m-technetium dimercaptosuccinic acid (99mTc-DMSA), creatinine levels, and follow-up time. Modified Schwartz formula was used to calculate eGFR using available data for height and serum creatinine measurements before and after open surgical intervention (constant k × height in centimeters/serum creatinine in micromoles per liter). The value of k was 0.413^5^. Urinary tract infection (UTI) diagnosis was defined as pyuria (over 5 white blood cells per high-power field), and a positive urine culture (i.e. >105 colony-forming units per milliliter of a microorganism). Hypercalciuria was defined if urinary calcium to creatinine ratio (mg/mg) was greater than the 95th percentile values for age (0-6 months 0.8, 7-12 months 0.6, and older than 12 months 0.2). Hyperuricosuria is diagnosed by examining urine uric acid concentration factored by creatinine clearance (urine_urate_ × serum_creatinine_ /urine_creatinine_, each in mg/dL); hyperuricosuria is defined by a value greater than 0.56 mg/dL of glomerular filtrate 6. Stones smaller than 3 mm in diameter were defined as microcalculi, and those 3 mm or larger in diameter were defined as macrocalculi^7^.

The data were processed and analyzed using the STATA MP/11 statistical package. Normality assumption was checked by Shapiro Wilk test. These variables were summarized as count (percentage), mean, standard deviation, and minimum and maximum values. Student t-test was used to compare eGFR levels before and after surgery. Statistical significance was accepted when the p value was less than 0.05.

## RESULTS

A male predominance was prominent with a male to female ratio of 3.3. The mean age at diagnosis was 20.2 ± 1.69 (4-60) months and the mean follow-up time was 54.6 ± 2.97 (7-100) months. Clinical and laboratory features of patients are given in Table 1. Nine patients had parental consanguinity. A family history for urolithiasis was found in nine patients. The mean quantitative cystine excretion of patients was 50,249 (593-23,658) µmol/gr creatinine. Ten patients had stones in the upper urinary system, three had stones in both the upper and lower urinary system. Ten patients had macrocalculi and three had microcalculi. In all patients, the stones were multiple. Stone analysis was performed in eight patients. Four of the stones were cystine, two were calcium stones and two were complex stones. Six patients had hydronephrosis. One patient had crossed fused renal ectopia. Renal outcomes of patients with cystinuria are shown in Table 2. Recurrent urinary tract infections were seen in only one patient. There was no vesicoureteral reflux on the voiding cystourethrography of the patient. Medical therapy, which consisted of over hydration, urine alkalization, captopril and/or tiopronin, was given to all patients. Nephrotic syndrome occurred in one patient as an adverse reaction to tiopronin. The mean estimated glomerular filtration rate (eGFR) of patients at last visit was 110 (77-156) mL/min/1.73m^2^. Surgical intervention was required in ten patients. Two patients (Patients 4 and 6) underwent two surgical procedures due to recurrence of stones. The other eight patients required only one surgical intervention. There was no significant difference between eGFR levels before and after surgical intervention (mean 92 *versus* 106, *p* = 0.36).

**Table 1:** Clinical and laboratory features of patients

Patient	Age of diagnosis (Months)-Gender	Presentation	Parental consanguinity	Family history	Hyper-calciuria	Hyper- uricosuria	Urinary Cystine (µmol/g/Creatinine)
1	22 - male	Abdominal pain,	[-]	[+]	[-]	[+]	5406
2	15 - male	Dysuria	[+]	[+]	[-]	[+]	3943
3	8 - male	Restlessness	[+]	[-]	[-]	[+]	1890
4	60 - male	Hematuria, dysuria	[-]	[-]	[-]	[-]	593
5	45 - male	Dysuria	[+]	[+]	[-]	[-]	5423
6	22 - male	Vomiting	[+]	[+]	[-]	[-]	6740
7	4 - male	Hematuria	[+]	[+]	[-]	[+]	4086
8	36 - male	Abdominal pain	[-]	[-]	[-]	[-]	2876
9	7 - male	Hematuria, dysuria	[-]	[-]	[-]	[-]	1875
10	19 - female	Vomiting	[+]	[+]	[-]	[+]	2941
11	5 - male	Restlessness	[+]	[+]	[-]	[-]	6811
12	8 - female	Hematuria	[+]	[+]	[-]	[-]	4299
13	6 - female	Restlessness	[+]	[+]	[-]	[-]	1523

**Table 2:** Renal outcomes of patients with cystinuria

Patients	Renal insufficiency due to stone obstruction	Recurrent urinary system infection	Renal scarring	Surgical treatment required	Relapses after surgery	Stone formation in the last visit
1	[-]	[-]	[-]	[+]	[-]	[-]
2	[-]	[-]	[+]	[+]	[+]	[-]
3	[-]	[-]	[+]	[+]	[-]	[-]
4	[+]	[-]	[+]	[+]	[+]	[-]
5	[-]	[-]	[+]	[+]	[-]	[-]
6	[-]	[-]	[+]	[+]	[+]	[-]
7	[-]	[-]	[-]	[-]	[-]	[+]
8	[-]	[-]	[-]	[+]	[+]	[+]
9	[-]	[-]	[+]	[-]	[-]	[+]
10	[-]	[+]	[+]	[+]	[-]	[-]
11	[-]	[-]	[-]	[-]	[-]	[-]
12	[-]	[-]	[+]	[+]	[+]	[-]
13	[-]	[-]	[+]	[+]	[+]	[+]

## DISCUSSION

Cystinuria, which has devastating effects on kidneys, consists of renal function impairment and nephrectomy^6^^,^ 8 ^,^ 9. Even with medical management, long-term outcome is poor due to insufficient treatment efficacy and low patient compliance^2^. Additionally, multiple urological interventions are required for most patients with cystinuria during their lifetime despite preventive measures. Assimos *et al*. revealed that open surgery for stone removal and nephrectomy were associated with an increased serum creatinine^4^. In the present study, only two patients underwent recurrent surgical interventions for recurrent urolithiasis and none of the patients required nephrectomy, which could have led to deterioration of renal function of the patients. Stone obstruction is an important surgical indication for cystinuric patients. Only one patient required surgery for renal impairment due to stone obstruction, improving the condition of the patient. If renal impairment secondary to stone obstruction were seen in more patients, renal function would have been better after surgery.

Combined metabolic abnormalities are frequently observed in patients with cystinuria. Shen *et al*.^10^ reported that hypercalciuria (46.7%), hyperoxaluria (40%), and hypocitraturia (33.3%) were the three most common metabolic abnormalities in patients with cystine stones in China. Sakhaee and colleagues^11^ reported hypercalciuria in 19%, hyperuricosuria in 22%, and hypocitraturia in 44% of 27 patients with cystine stones. In the present study, hyperuricosuria was the most common metabolic abnormality in patients with cystinuria. This result may be related to dietary differences of various populations, such as the relatively high protein and low vegetable intake of most of our patients. Urinary oxalate and citrate levels could not be evaluated because of lack of these tests in our center.

In developing regions such as various areas of Turkey, Iran, India, China, Indochina, and Indonesia, where people are fed on carbohydrate rich but low-protein diets, bladder stones are seen commonly. In contrast, upper urinary tract stones are common in the developed countries^12^. In a study conducted in Turkey, stones were localized in the bladder in nearly half of the patients^13^. However, in recent studies, stones in the upper urinary tract were reported to be more common in Turkey^14^^-^ 16. Celiksoy *et al*.^16^ reported that in cystinuric patients multiple lower urinary tract stones and recurrence were significantly higher. In the present study, most of the patients' stones were in the upper urinary tract. This difference also may be attributed to different patterns of diet in Turkey. All patients had multiple stones and recurrence ratio was high, which is consistent with the literature.

Cystinuric patients often have mixed calculi composed of substances other than cystine that can disguise the presence of cystinuria and delay the diagnosis even many years after the onset of initial symptoms^17^. Resnick *et al*. reported that heterozygosity for cystinuria predisposes to calcium oxalate stone formation^18^. In another study, all patients with homozygous cystinuria developed pure cystine stones. Calcium oxalate, uric acid, and cystine stones formed in 51.4, 20.0, and 8.6% of the patients with heterozygous cystinuria, respectively^19^. In our series, only four of the stones were pure cystine. Although hypercalciuria was not detected in the patients, two patients developed calcium stones. Genetic analysis of the patients could not be performed due to unavailability of the genetic tests in our center. These differences may be attributable to genetic defects (homozygous or heterozygous) and dietary differences of high protein and low vegetable content.

In one patient, nephrotic syndrome developed due to tiopronin treatment. His renal biopsy was consistent with minimal change disease. He was treated with captopril instead of tiopronin and steroid for nephrotic syndrome. Other reports also describe nephrotic syndrome in cystinuria treated with tiopronin^20^. Ferraccioli *et al*.^21^ reported the biopsy findings of six patients who developed nephrotic syndrome during treatment with tiopronin. The majority of patients had membranous glomerulonephritis, while one patient had mesangioproliferative glomerulonephritis and another one had glomerulonephritis with segmental deposits in the mesangium.

Although renal stones are an infrequent cause of renal failure, some lithiasic forms present a greater risk, such as in hereditary stone diseases (e.g. cystinuria, primary hyperoxaluria, Dent's disease), primary struvite stones, and infection-related urolithiasis associated with anatomic and functional urinary tract anomalies and spinal cord injury. Recurrent bouts of obstruction and/or crystal-specific biological effects on tubular epithelial cells and interstitial renal cells may activate the fibrogenic cascade responsible for the loss of renal parenchyma. In clinical terms, frequent stone relapses, episodes of urinary tract infection and obstruction, number of urological interventions, and size of the stone are all significantly associated with the risk for renal failure^22^.

In our sample, only one patient developed renal failure due to stone obstruction and percutaneous nephrostomy was performed. Although recurrent urinary tract infections were seen in only one patient, renal scarring was detected in nine, and eight of these patients required surgical intervention. Thus, it can be considered that renal scarring developed due to crystal-specific biological effects on tubular epithelial cells rather than recurrent urinary tract infection.

The limitations of this study are the retrospective design, limited number of patients, and lack of genetic and metabolic tests (oxalate and citrate in urine samples).

In conclusion, cystinuria is a condition that requires life-long monitoring, dietary constraints, pharmacologic prevention, and frequent surgical intervention. Surgical intervention does not have a negative impact on renal functions in cystinuria. Therefore, in patients with cystinuria who are not responsive to medical treatment, surgical intervention is a safe and efficient method to remove the stones.
